# Terminal osseous dysplasia with pigmentary defects (TODPD) due to a recurrent filamin A (FLNA) mutation

**DOI:** 10.1002/mgg3.90

**Published:** 2014-08-08

**Authors:** Nicola Brunetti-Pierri, Maria Torrado, Maria del Carmen Fernandez, Ana Maria Tello, Claudia L Arberas, Antonella Cardinale, Pasquale Piccolo, Carlos A Bacino

**Affiliations:** 1 Telethon Institute of Genetics and MedicineNaples, Italy; 2 Department of Translational Medicine, Federico II University of NaplesNaples, Italy; 3 Servicio de Genética, Hospital GarrahamBuenos Aires, Argentina; 4 Hospital de Niños Dr. Ricardo GutiérrezBuenos Aires, Argentina; 5 Department of Molecular and Human Genetics, Baylor College of MedicineHouston, Texas

**Keywords:** Filamin A, FLNA, terminal osseous dysplasia with pigmentary defects, X-linked dominant

## Abstract

Terminal osseous dysplasia with pigmentary defects (TODPD) is an X-linked dominant syndrome with distal limb anomalies, pigmentary skin defects, digital fibromas, and generalized bone involvement due to a recurrent mutation in the filamin A (*FLNA*) gene. We here report the mutation c.5217G>A in *FLNA* in three families with TODPD and we found possible germline and somatic mosaicism in two out of the three families. The occurrence of somatic and germline mosaicism for TODPD indicates that caution should be taken in counseling recurrence risks for these conditions upon presentation of an isolated case.

## Introduction

Terminal osseous dysplasia with pigmentary defects (TODPD; OMIM 300244) is an X-linked dominant male-lethal disorder with pigmentary anomalies of the skin, generalized skeletal abnormalities, mainly involving the limbs, digital fibromas, multiple oral frenulae, iris colobomas, cardiac, and urogenital malformations (Bacino et al. 2000; Brunetti-Pierri et al. 2010). So far, a unique, recurrent filamin A (*FLNA*) gene mutation was found to be responsible of TODPD by X-chromosome exome sequencing (Sun et al. 2010). The same mutation c.5217G>A, affecting the last nucleotide of exon 31 of the *FLNA* gene, has been found in six unrelated cases of TODPD (Sun et al. 2010). The mutation activates a cryptic splice site, removing the last 48 nucleotides from exon 31, results in a loss of 16 amino acids at the protein level. In the three studied families, the mutation was found to segregate with the disease, and it was transmitted from the affected mothers to the affected daughters (Sun et al. 2010). Because of nonrandom X-chromosome inactivation, the mutant allele is not expressed in patient fibroblasts and expression of aberrant RNA could only be detected in cultured fibroma cells from surgical material (Sun et al. 2010).

We here report the *FLNA* mutation in three families with TODPD and we found possible germline and somatic mosaicism in two of them. Clinical findings of newly described pedigree 1 and pedigree 2 are summarized in Table1.

**Table 1:** Summary of clinical features

Clinical findings	Pedigree 1			Pedigree 2	
	II.2	II.4	I.2	II.2	I.2
Pigmentary spots of the face	+	+	−	+	+
Oral frenulae	+	+	+	+	+
Digital fibromas	−	+	−	+	−
Hand abnormalities	+	+	−	+	+
Foot abnormalities	+	+	−	+	−
Short stature	−	−	−	−	+
*FLNA* mutation c.5217G>A on blood DNA	+	+	−	+	−

### Pedigree 1

**Figure 1:**
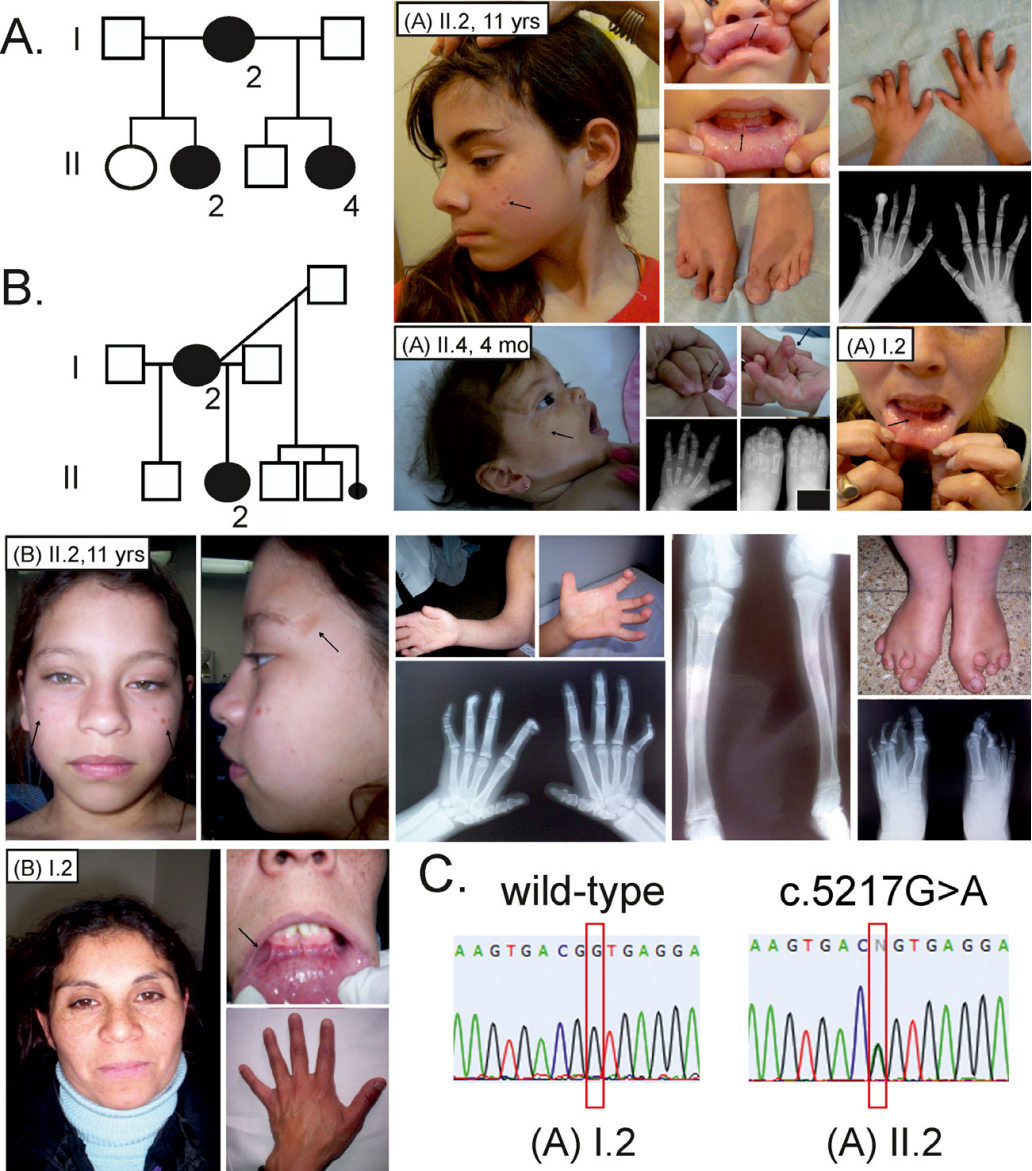
(A) Pedigree 1 family. Patients II.2 and II.4 are depicted. Note facial hyperpigmented spots, atrophic lesions of the temporal region, oral frenulae, brachydactyly and camptodactyly, and digital fibromas (arrows). Feet of patient II.2 showed variable shortening of second through V toes, with elongated first toes bilaterally. Hand X-rays of patient II.2 showed in the left hand a short and wide III metacarpal, thin IV metacarpal, short V metacarpal, mild hypoplasia of all middle phalanges, hypoplasia of all distal phalanges, fusion of the III carpal-metacarpal, joint angulation; in the right hand thin II metacarpal, slight hypoplastic tufts. The thumbs are spared. The face of patient II.4 shows atrophic lesions with hyperpigmentation (arrow). Hands of patient II.4 have periungueal fibromatosis lesions over the fourth digit (arrows). Hand X-rays of patient II.4 show delayed carpal center ossification; the III metacarpal is hypoplastic and widened (bullet-shaped); angulated middle phalanges-distal phalanges joint of the III digit is noted. Foot X-rays of patient II.4 show asymmetrical involvement; on the right foot the II and IV metatarsals are hypoplastic or have an amorphous shape, the great toes are elongated. On the far right, a picture of both girls' mother is shown (I.2) with multiple frenulae in the lower lip (arrow). (B) Pedigree of family 2 is shown. Frontal and profile views of the face of patient II.2 with hyperpigmented atrophic lesions over malar and temporal regions (arrows). Right arm showed mild pterygia and brachydactyly on both hands mostly affecting first and third through V metacarpals. Camptodactyly is also present. On the lower figure, individual I.2 is shown. Note her multiple frenulae (arrow) and brachydactyly. (C) Sanger sequencing shows the c.5217G>A mutation in the index case (II.2 from pedigree 1 [A]) and normal sequence in her mother (I.2).

Two affected half sisters born from different fathers presented with dysmorphic features, pigmentary spots of the face, hand and foot abnormalities. The oldest affected female (II.2) had a normal sister, while the youngest girl (II.4) had a normal brother. There was neither reported consanguinity nor history of miscarriages (Fig.1A). The oldest child (II.2) was born to an uneventful pregnancy by normal vaginal delivery after 42 weeks of gestation with a birth weight of 2590 g. She had a normal psychomotor development. She had some difficulties with math and was held back in the first grade and at 11 years she was attending the fifth grade. At the time of evaluation she was 11 years and 4 month-old and her height was 144 cm (25th–50th centile), weight 35 kg (25th–50th centile), and head circumference 50 cm (10th centile). On exam, she showed hypotrophic skin lesions in the parietal regions, adjacent to the orbits and on both cheeks, and mild micrognathia. By report, she had numerous frenulae that were surgically resected. There was a delayed eruption of permanent dentition. The left hand showed camptodactyly of the II and IV digits, hypoplasia of the III digit, hypoplasia of the V digit with ulnar deviation, as well as ulnar deviation of the thumb. The right hand had similar camptodactyly of II and IV digits with ulnar deviation and hypoplasia of the III digit and hypoplasia and clinodactyly of V digit. Limb anomalies also included hypoplasia of III–V toes more severe on the IV toe of the right foot, hypoplasia of II–V metatarsals, syndactyly between II and III toes on the left foot, and elongation of first toes bilaterally (Fig.1, subject [A] II.2). She also exhibited marked lumbar lordosis, thoracic dextroscoliosis, and winged scapulae.

The youngest affected sibling (II.4) was born to a 38 week gestation by cesarean section with a birth weight of 2850 g. She was evaluated at 4 months of age and at that time, the physical exam showed a length of 58.4 cm (10th–25th centile), weight 3300 g (<3rd centile), and head circumference 39.5 cm (10th–25th centile). She had anteverted nares, short philtrum, multiple oral frenulae, atrophic lesions over the temporal regions and cheeks, and a bright hyperpigmented spot over the right shoulder. She also has a flat hemangioma of the tip of the nose. There were periungueal fibromatous lesions on both IV fingers. The right hand showed generalized brachydactyly more marked for phalanges I and V, clinodactyly of V finger, and ulnar deviation of II and IV digits. Both feet had toe hypoplasia involving mainly II, IV, and V toe bilaterally, as well as tibial deviation of both I toes. Both first toes were elongated. She had syndactyly between IV and V toes on the right foot and overlapping toes on the left foot (Fig.1, subject [A] II.4).

Physical exam of the mother of the two affected sisters was only remarkable for multiple frenulae in her lower lip (Fig.1, subject [A] I.2) whereas the rest of her exam was normal with no skin findings or limb deformities.

### Pedigree 2

The index case is an 11-year-old female who presented with pigmentary spots of the face, multiple oral frenulae, and abnormalities of the hands and feet (Fig.1, subject [B] II.2). The patient was born to a pregnancy complicated by an attempted termination that led to profuse bleeding necessitating hospitalization. She was ultimately born full term with a birth weight of 2250 g by cesarean section performed because of cephalo-pelvic disproportion. She developed respiratory distress at birth that required oxygen via an oxyhood. She had delays in acquisition of both gross and fine motor milestones. Her mother had a total of three healthy sons with two previous partners, as well as a first trimester miscarriage (Fig.1B).

On physical exam, she had normal growth parameters. Her forehead had a square appearance, flat supraorbital ridges, upslanting palpebral fissures, left ptosis with distichiasis on the same side, depressed nasal bridge, wide nose base with a round tip of the nose, and full fleshy lips (Fig.1, subject [B] II.2). The mouth showed multiple frenulae superiorly and inferiorly, malocclusion, conical shape teeth, and mild micrognathia. She exhibited skin lesions over the face with hyperpigmented and atrophic appearance, mostly in the lateral aspects of the forehead. The extremities showed limited elbow mobility with pterygium of the arms. The hands showed brachydactyly, hypoplasia of the first metacarpals, camptodactyly of II through V digits with symphalangism involving the distal interphalangeal joints, clinodactyly of the V fingers, and abnormal flexion creases. There was generalized digital fibromatosis. There was also tibial bowing of the legs and the feet showed bilateral hallux valgus, hypoplasia of the II metatarsals, and brachydactyly of III through V toes bilaterally (Fig.1, subject [B] II.2). A 2 mm skin punched biopsy obtained from the forehead lesions showed epidermis with flattening of the epidermic layer and mild increase in connective tissue, and normal adnexa. A 4 mm fragment of an oral mucosal appendage showed hyperkeratosis, acanthosis, papillomatosis, and spongiosis, with minimal lymphocytic infiltrate leading to the diagnosis of squamous papilloma.

Her mother had short stature (4SD below the mean), pigmentary lesions in her face, multiple frenulae, right eye distichiasis, and brachydactyly (Fig.1, subject [B] I.2).

### Pedigree 3

Clinical descriptions of affected members from pedigree 3 have been previously reported (Bacino et al. 2000; Brunetti-Pierri et al. 2010).

### FLNA sequencing

Both daughters of pedigree 1 were found to carry the c.5217G>A in *FLNA* gene, while the mutation was not detected in the DNA extracted from blood in the mother (Fig.1C and Table1).

The affected daughter of pedigree 2 was found to carry the c.5217G>A in *FLNA* gene, while the mutation was not detected in the DNA extracted from blood in the mother (Table1).

One of the affected patients of pedigree 3 was screened for *FLNA* mutations by complete Sanger sequencing (Brunetti-Pierri et al. 2010). However, the mutation was missed at the initial screening and following the publication of the article reporting the recurrent *FLNA* mutation in TODPD (Sun et al. 2010), we resequenced the index case and the c.5217G>A was indeed detected. Two additional affected female patients of the family were also found to carry the c.5217G>A mutation.

## Discussion

*FLNA* gene is involved in signaling pathways that mediate organogenesis in multiple systems including the skeleton. As a consequence of such pleiotropic functions, mutations in *FLNA* gene result in a spectrum of disorders affecting the central nervous system, the cardiovascular system, and the skeleton (Robertson 2004). In this study, we confirm in three additional families, including the original reported pedigree (Bacino et al. 2000), that a recurrent mutation in *FLNA* gene results in TODPD, a newly recognized *FLNA* disorder.

Although digital fibromas and pigmentary anomalies of the skin are distinctive features of TODPD and not of other *FLNA*-related disorders, the generalized bone involvement including bowing, mesomelic shortening, abnormal bony texture, the narrow iliac wings and S-shaped tibias (Brunetti-Pierri et al. 2010), observed in TODPD, are also present in Melnick-Needles syndrome and otopalatodigital (OPD) syndrome that are also caused by *FLNA* mutations (Robertson et al. 2003).

Skewed X-inactivation is observed in females heterozygous for *FLNA* Melnick-Needles syndrome and OPD mutations, suggesting that cells need normal FLNA to survive. Previous studies have shown that mutated FLNA have increased actin-binding affinity supporting a gain-of-function mechanism (Clark et al. 2009). The correct interaction of FLNA with F-actin is important for the structural stability of the cytoskeleton and for normal signal transduction (Hartwig et al. 1980). The TODPD mutant *FLNA* allele was not found to be expressed in patient fibroblasts while it was expressed in cultured fibroma cells (Sun et al. 2010). At the protein level, c.5217G>A encodes the second-to-last amino acid of the immunoglobulin-like repeat 15, which is very close to the first hinge region that has been recently found to interact with meckelin, a protein defective in Meckel-Gruber syndrome (Adams et al. 2012), in addition to other ligands implicated in actin remodeling (Tu et al. 2003). It can be speculated that the c.5217G>A mutation specifically disrupts the interaction of FLNA with specific ligand(s) in skin fibroblasts thus altering the intracellular signaling and inducing cell proliferation and digital fibromas.

The interpretation of pedigree 1 suggests that the mother was a germline mosaic for the *FLNA* mutation. Gonadal mosaicism and likely low level somatic mosaicism is the most likely explanation for the finding of a healthy parent who has two females affected with an X-linked dominant disorder; and it is further supported by the physical exam showing multiple oral frenulae as an isolated finding. The mother in pedigree 2 instead presented short stature, multiple oral frenulae, and pigmentary lesions of the face suggesting the presence of both gonadal and somatic mosaicism.

Germline mosaicism is well documented in a wide range of genetic diseases with the phenomenon being commonly encountered in conditions such as osteogenesis imperfecta and Duchenne muscular dystrophy (Zlotogora 1998). To date, somatic and germline mosaics have been reported for only a few X-linked disorders, and remarkably, they have been reported in a pedigree with OPD syndrome due to mutations of *FLNA* (Robertson et al. 2006). Several individuals with germline mosaicism have also been shown to be low level somatic mosaics. In the instance described here (pedigrees 1 and 2), there were minimal clinical manifestations in the mothers of the female index cases with TODPD, but no tissues other than blood were available to investigate the presence of the mutation.

The description of possible germline mosaicism for the mutation c.5217G>A leading to TODPD in two sisters of pedigree 1 has important implications for molecular diagnosis interpretation and clinical evaluation of TODPD. Importantly, the possibility of germline mosaicism and/or low-level somatic mosaicism must now be considered in counseling recurrence risks for families with a single isolated individual with TODPD. The risk of recurrence when the mother has tested negative for the causative *FLNA* mutation is higher than the background new mutation rate.

In conclusion, a number of the perplexing features of the genetics of the TODPD have now been resolved: (1) the disease is caused by a mutation in the *FLNA* gene; (2) so far only one recurrent mutation has been reported in a total of nine unrelated cases; (3) mothers with minor or no signs of the disease may present somatic and germline mosaicism.

## Conflict of Interest

None declared.
